# Legitimating Corporate Power: Shareholderism versus Stakeholderism

**DOI:** 10.1093/ojls/gqaf037

**Published:** 2025-11-22

**Authors:** Heikki Marjosola

**Keywords:** corporate governance, corporate law, legitimacy, corporate social responsibility, shareholder primacy, stakeholder theory

## Abstract

This article assesses the legitimising strategies of ‘shareholderism’ and ‘stakeholderism’ through the dual lenses of input and output legitimacy widely used in political theory. Here, output legitimacy evaluates corporate decision making by its contribution to societal welfare, whereas input legitimacy requires that corporate decisions reflect the preferences of its legitimate stakeholders. On both fronts, shareholderism and stakeholderism offer incomplete strategies to legitimise corporate authority. Shareholderism, although firmly grounded in state-derived legitimacy, fails to address certain structural problems, such as corporate political power and the geographic mismatch between jurisdictions and capital. Most stakeholderists, by contrast, would have corporate leaders make distributive judgments in place of the majoritarian, yet ineffective, political process. In terms of output legitimacy, their alternative is plausible, but they fail to engage with the participatory requirements of input legitimacy. The emerging proceduralist agenda, drawing on principles such as transparency and stakeholder engagement, offers a thin basis for corporate legitimacy.

## 1. Introduction

The debate between those who believe that corporations exist to maximise shareholder wealth (shareholderists) and those who think that corporations serve a broader societal purpose and should therefore pursue the goals of a more diverse gallery of stakeholders (stakeholderists) has reached another impasse. Shareholderists insist that shareholder primacy is the best way to maximise both shareholder value and social welfare, whereas putting stakeholders on a par with shareholders would damage shareholders as well as societies, and would in any case be unworkable as law. For shareholderists, societal problems such as inequality or climate change inhabit the world of external economies, best addressed by targeted measures outside corporate law proper. Stakeholderists, in turn, criticise shareholderists for underestimating the transformative potential of corporate law reform and for overestimating the effectiveness of regulation. Even if appropriate rules were in place, they claim, corporations would find a way to neutralise or avoid them. Therefore, corporations should be reformed from within. They are, after all, creations of law.^1^

This article draws on normative democratic theory to make sense of the perennial and arguably sterile corporate purpose debate.^2^ Similar approaches have been taken before. Observing the equally arrested Corporate Social Responsibility debate in the late 1970s, David Engel argued that debating the topic is possible only ‘against the background of a general political theory’.^3^ Following his lead, Roberta Romano constructed an idealised typology of democratic ideals to link corporate law reform proposals to the reformists’ implicit political beliefs.^4^ Both Engel and Romano criticised the reformists of their time for placing excessive focus on the *means* of reform while failing to articulate their *ends*, namely ‘the vision of the good society’ that underpinned their policy programmes.^5^

In a similar spirit, this article analyses the shareholderism versus stakeholderism controversy through the lens of corporate legitimacy, asking what types of strategies both sides of the debate advocate to legitimise corporate power, and how these strategies link with their broader political visions—whether explicitly articulated or not. The theme is increasingly topical as corporate governance is emerging as ‘a battleground for contrasting views of legitimacy’.^6^ It is appealing also because the analytical point of departure appears to be one of agreement, rather than disagreement. The argument that large corporations are responsible for significant and uncompensated social costs, often made by advocates of stakeholderism, is now admitted even by their most prominent adversaries. As Lucian Bebchuck and Roberto Tallarita note:

There is a widespread and growing recognition that, although corporations have been a major engine for growth, their profit-seeking operations contribute to a wide array of society's problems and impose serious negative externalities on employees, communities, consumers, and the environment.^7^

The problem of corporate legitimacy, therefore, stems from corporate power. The choices of corporations as economic, social and political actors affect the environment and the general community in countless ways.^8^ This finding is no less controversial today than it was in ancient Rome.^9^ Such powers, unlike market transactions, which some argue have ‘intrinsic legitimacy’, require legitimation.^10^

The article evaluates the legitimation strategies of shareholderism and stakeholderism against a normative standard of democratic legitimacy widely used in European and international governance studies. In democratic theory, the concepts of input and output legitimacy have been used to distinguish between governing effectively *for the people* (output legitimacy) and enabling participation *by the people* (input legitimacy).^11^ Adapting these concepts to corporate decision making, output legitimacy here refers to the quality or efficiency of corporate decision making based on some measure of social welfare or ‘common good’. Input legitimacy, in contrast, posits a procedural criterion according to which decisions of corporate leaders are legitimate only if they reflect the authentic preferences of those who are affected by them, that is, the corporation’s legitimate stakeholders.^12^

Much of the existing normative commentary on corporate law and governance can be characterised and evaluated in terms of input and output legitimacy. For example, Melvin Eisenberg draws on *output legitimacy* when he argues that the legitimacy of the corporate system can be based on the belief that ‘placing control of the factors of production and distribution in the hands of privately appointed corporate managers … achieves a more efficient utilization of economic resources than that achievable under alternative economic systems’.^13^ In his famous *New York Times* manifesto, Milton Friedman draws primarily on *input legitimacy*. Corporate executives should not be viewed as civil servants, he argues, unless there exists a political process for selecting them. Otherwise, there would be no way to ensure that the managers’ decisions about ‘whom to tax by how much and for what purpose’ respect the preferences of those affected.^14^ For Edward Rock, such lack of accountability represents ‘the fundamental problem of political legitimacy’.^15^

The article finds that neither shareholderism nor stakeholderism sets forth a convincing legitimation strategy based on the input/output criteria. The intellectual foundations of shareholderism are conceptually compatible with the established liberal-democratic model. Deriving its legitimacy from the state, shareholderism maintains a sharp divide between output-oriented efficiency (the economic system) and input-oriented distribution (the political system). However, shareholderism fails to address serious structural problems, such as the corporate political power and the geographic mismatch between jurisdictions and capital, which prevent governments from effectively performing their rule-setting functions. Without credibly addressing negative externalities and monopoly issues, shareholderism fails to meet the requirements of both input legitimacy (stakeholder interests) and output legitimacy (social welfare).

Most stakeholderists, in contrast, would have corporate management—insulated from both shareholder and stakeholder control—make ‘common good’ oriented distributive judgments in place of the majoritarian, yet ineffective, political process. In terms of output legitimacy, their vision appears plausible, although the political philosophies or democratic ideals underpinning contemporary stakeholderism remain, at best, rudimentary. Nevertheless, stakeholderism fails to engage with the participatory requirements of input legitimacy. The emerging proceduralist agenda, which legitimises the transformation of corporations into distributive polities by drawing on principles such as stakeholder participation and transparency, offers but a thin foundation for corporate legitimacy. The analysis concludes with a tentative critique of ‘the deliberative turn’ in corporate governance.

The article proceeds as follows. The following section considers certain established notions of corporate legitimacy, highlights their shortcomings and argues for the use of input and output legitimacy as a valuable normative framework for evaluating the legitimising strategies of shareholderism and stakeholderism. The substantive analysis of the contemporary corporate objective debate is carried out in sections 3 (shareholderism) and 4 (stakeholderism). Section 5 concludes.

## 2. Corporate Power and Legitimacy

### A. Corporate Legitimacy beyond Legal Validity

The topic of corporate legitimacy touches upon a fundamental theoretical controversy within corporate legal scholarship. What does any notion of political legitimacy have to do with a *private entity* whose existence ultimately depends on freedom of contract and individual autonomy?^16^ As is often pointed out, even John Rawls kept the realm separate from his political philosophy.^17^ Whether corporations are public or private in character has indeed been one of the most crucial issues in corporate legal theory.^18^ This debate cannot, and need not, be revisited here. Suffice it to say that, just as is the case with the corporate objective debate, the public/private controversy is unintelligible without political theory since the adopted definitions themselves express different views of ‘the nature and purpose of our social life’.^19^

Beyond such legal-conceptual debates, corporate legitimacy has been the subject of a sprawling discourse. In an interdisciplinary analysis, James Brummer identifies eight different legitimation strategies or models of institutional legitimacy for corporations, while also adding his own.^20^ Such diversity reflects the ‘notoriously elusive’ nature of the legitimacy concept—even in political science.^21^ Most would agree that the legitimacy problem is somehow linked to ‘the normative dimension of power relations and the ideas and practices that give those in power their moral authority and credibility’.^22^ But there is no agreement on what criteria we should employ to determine whether power is rightfully obtained and exercised, and whose judgment counts in deciding when those criteria are met. The answer often depends on one’s professional orientation, specifically whether the standpoint is that of a moral or political philosopher, or a social or political scientist.^23^

Lawyers habitually equate legitimacy with legal validity: if power is acquired and exercised in conformity with law, it is considered legitimate.^24^ Such formal legal legitimacy has been the predominant concern of corporate law scholarship as well.^25^ The shortcomings of this view are apparent. First, while congruence with *social rules* is an important element of any normative notion of legitimacy, not all social rules, eg those of a customary or conventional nature, are part of a formal legal order. Actions outside law are not automatically ‘just’ or ‘legitimate’.^26^ What is more, formal legal rules also require normative validity. As Beetham writes,

disputes about the legitimacy, or rightfulness, of power are not just disputes about what someone is legally entitled to have or to do; they also involve disagreements about whether the law itself is justifiable, and whether it conforms to moral or political principles that are rationally defensible.^27^

This means that the corporation cannot derive its legitimacy from the political and legal system, as suggested, for example, by Eric Orts,^28^ unless the system itself and the rules it produces satisfy some normative criteria of legitimacy. A legal system enabling corporations to externalise costs on unconsenting third parties will struggle to meet many normative criteria, including ‘wealth maximisation’, which Richard Posner defends as the appropriate norm for social and political choices. As he explains, the domain of this criterion should be restricted ‘to actual markets that are free from serious problems of monopoly or externality’.^29^

A broader conceptualisation of legitimacy was introduced by James Willard Hurst in his 1970 essay.^30^ As any other social institution, he argued, a corporation must be legitimated by its usefulness to some social end ‘other than its own perpetuation’ (utility) and be ‘accountable to some judgement other than that of the power holders’ (responsibility).^31^ This provides a more nuanced understanding of legitimacy, particularly when accountability is understood to encompass both internal accountability (towards shareholders) and external accountability (towards legitimate stakeholders affected by corporate decision making).^32^ However, Hurst’s utility and responsibility criteria are difficult to distinguish analytically.^33^ Most importantly, no *democratic* notion of legitimacy can view utility, or institutional performance, as something that may compensate for lack of accountability.^34^ Evaluating performance against some notion of ‘utility’ (or ‘public interest’, ‘common good’, etc) requires procedures for identifying *what precisely is* in the public interest of a given constituency.^35^ Non-majoritarian technocratic institutions with specific expertise (eg central banks) may operate on the basis of ‘assumed citizen interests’ such as wealth maximisation because we may safely assume that ‘individuals will collectively prefer more economic welfare over less’.^36^ But to ensure accountability, only limited tasks not involving distributive judgments are typically delegated to such institutions.^37^ This, as noted above, is not the case with self-authorised corporations, whose actions affect peoples and the planet in multiple and unpredictable ways.

To conclude, a democratic conception of corporate legitimacy—at least a liberal-democratic one—cannot ignore the affected stakeholders’ preferences. These must somehow be expressed, and not taken as given, if they are to serve as inputs to processes of social choice.^38^ As Beetham argues, legitimacy should be understood as a multidimensional concept which comprises rules and socially accepted normative beliefs, but also appropriate actions of recognition and acknowledgement.^39^ The next section shows how the concepts of input and output legitimacy can be operationalised to integrate these aspects.

### B. Input and Output Legitimacy

The concepts of input and output legitimacy, as applied here, integrate two different yet closely connected notions of legitimacy. To reiterate, input legitimacy emphasises governing processes ensuring that decision making is responsive to the manifest preferences, values and concerns of legitimate stakeholders, whereas output legitimacy depends on effectiveness of corporate decision making, or its problem-solving capacity, in relation to some justified notion of social welfare or common good.^40^ The approach therefore combines two important strands of normative democratic theory: the strand predominantly dealing with procedural justifications of power structures and the consequentialist strand more concerned with their substantive outcomes.^41^ Of course, applying the concepts of input and output legitimacy is not the only way to combine these two aspects. In their recent normative analysis of corporate legitimacy, Stavros Gadinis and Chris Havasy also use both procedural and substantive criteria for establishing the ‘moral legitimacy’ of corporations.^42^

Drawing from such objective standards, this article falls under the normative genre of legitimacy studies. The approach should be distinguished from the empirical or sociological genre, which deploys concepts and tools of social science to measure social attitudes as a subjective measure of legitimacy.^43^ The two genres are connected, but separable.^44^

The concepts of input and output legitimacy have had a great influence within the discourse on European integration and the alleged democratic deficit of the European Union. However, they have also been applied in global and transnational governance studies to scrutinise the gradual shift of authority from public bodies to non-state institutions, which lack traditional democratic credentials.^45^ Within the broad realm of governance studies, no other legitimacy concept has been as useful and popular.^46^ This article is the first attempt at applying the input and output legitimacy criterion in the more general context of corporate governance.

Considering the input- and output-oriented dimensions together is fruitful because they are connected in a logical, albeit complex, way. Like political decisions, corporate decisions could be considered normatively valid because they were made by appropriate procedures (eg a shareholder resolution on a major transaction), or they might be valuable on independent grounds, regardless of the way they have been reached (eg because they increase profits and maximise the value of the corporation). But these dimensions are necessarily interlinked. Any concept of output value, such as profit, should ideally integrate the individual ends that the relevant constituents care about (profit? ). Without such a link, the procedures determining who gets to decide what would amount to nothing but an ‘empty ritual’.^47^ A democratic notion of ‘the public interest’ therefore requires governance mechanisms capable of making individual values, preferences and concerns visible to relevant decision makers. In other words, output legitimacy is an essential element of *democratic* governance and cannot be analytically separated from input legitimacy.^48^ They must be combined to make corporate decision makers exposed to the articulated interests of affected stakeholders.^49^

At the same time, the analytical relationship between input and output is not straightforward. In many contexts, as in network-based global governance, increasing input legitimacy by enabling participation of affected groups may automatically improve outcomes and thus increase output legitimacy.^50^ But this is not always the case. Referenda, for example, maximise citizen input but may produce suboptimal results on the output side.^51^ Such questions have their obvious parallels in corporate governance literature (‘does increasing shareholder/stakeholder voice improve firm performance?’).

The remainder of this article assesses the normative models of shareholderism and stakeholderism against this standard of democratically legitimate decision making.

## 3. Shareholderism

Renée Adams, Amir Licht and Lilach Sagiy, who first introduced the term, define shareholderism as an ‘ideology-like stance’, which involves ‘a motivated, principled approach that generally considers it a desirable strategy to enhance shareholder value’.^52^ Corporations should therefore be organised and run with a view to advancing the interests of shareholders, while other stakeholders such as employers, creditors, customers and communities have instrumental value only.^53^ The focus here will be on the agency cost tradition originating from the Chicago school. The principal-agent model has become the dominant theory of the firm and lends modern corporate law its ‘economic structure’.^54^ Other shareholderist approaches, such as those advocating the benefits of managerial power or board authority as opposed to the ideal of full accountability to shareholders, will not be considered here.^55^

The section starts with the output-oriented justifications of shareholderism, exploring arguments relating to efficiency and social welfare. The second subsection focuses on input legitimacy, asking what type of mechanisms shareholderism prescribes to guarantee that corporate decision making reflects the preferences of the affected stakeholders. The section finishes with a critical evaluation of the proposed legitimation strategies of shareholderism.

### A. Output Legitimacy of Shareholderism

The agency cost model presents a straightforward efficiency explanation for the existing corporate law system. For Chicago economists such as Michael Jensen, the entire question whether the interests of shareholders should be prioritised over other constituencies is framed incorrectly. The real issue is ‘what firm behavior will result in the least social waste—or equivalently, what behavior will get the most out of society’s limited resources’.^56^ The appropriate objective of corporations is therefore not shareholder value, but ‘total market value’, defined as ‘the sum of the market values of the equity, debt, and any other contingent claims outstanding on firm’.^57^ This is also what corporate law should aim for.^58^

What, then, makes shareholders special? The short version of the Chicago answer is that shareholders, with residual claims and rights to net cash flows, are best placed to mitigate agency costs, which arise because writing and enforcing contracts among participants to the corporate endeavour is not costless.^59^ For instance, corporate directors’ fiduciary duties of care and loyalty are owed to shareholders because the contract between directors (as agents) and shareholders (as principals) is more incomplete than the other contracts that constitute the corporation. Stakeholders such as employees, suppliers and creditors are better positioned to protect themselves by specified contractual terms or via influencing law making. Fiduciary duties therefore provide an efficient, market-mimicking solution to a specific contracting problem.^60^ The objective of profit maximisation in no way contradicts stakeholder interests since equity is the surplus that remains after all other contingent claims have been satisfied. Because of this, shareholders also have the appropriate incentives to take risks and monitor management. Finally, shareholderism maintains that managing for all stakeholders would be inefficient. Multiple objectives would increase agency costs and destroy value because corporate leaders cannot be assessed or held accountable without clear criteria for performance.^61^

Consequently, corporations maximise output best by focusing on the interests of shareholders as residual risk bearers. In this purely positive context, shareholderism—defined as an ‘ideology-like stance’—is a misnomer. The large public corporation with separated ownership and control is just a form of economic organisation that has triumphed in a process of natural selection. There is no normative commitment to any form of organisation or capital structure: the ‘liberal legitimacy’ of the corporation rests solely on its efficiency.^62^ Accordingly, law should enable its eclipse by a more efficient organisational form, if such exists. The job of corporate law is to enable contracting by mimicking market transactions; the rest is up to the markets.^63^

The contractarian theory of corporation nevertheless rests on certain assumptions with broader normative implications. Most importantly, shareholderism maintains that it also promotes social welfare. As the standard argument goes, the goal of maximising shareholder value is merely an intermediate goal, ‘the best means by which corporate law can serve the broader goal of advancing overall social welfare’.^64^ The appropriate normative goal of corporate law is accordingly ‘to advance the aggregate welfare of all who are affected by a firm’s activities, including the firm’s shareholders, employees, suppliers, and customers, as well as third parties such as local communities and beneficiaries of the natural environment’.^65^

The obvious problem with this output-legitimating function of corporate law is that not everyone affected by a firm’s activities are part of the corporate nexus of contracts. How, then, is aggregate (social) welfare connected to corporate output? The shareholderist answer is simple: the ‘equity surplus’ equals the added value of the corporate enterprise also in terms of social welfare.^66^ Most accounts accordingly focus on the effective implementation of shareholder value without scrutinising its underlying legitimacy.^67^

For shareholderists, social welfare is therefore ‘a loose metaphor’ rather than ‘a precise yardstick’.^68^ But it is usually acknowledged that the welfare-maximising promise of for-profit corporations holds only in the absence of monopolies or significant externalities.^69^ If corporations have enough market power to cut production and raise prices at will, shareholder wealth maximisation does not translate into social wealth.^70^ And where there are significant externalities, public interventions may be needed to impose social costs on the firm’s bottom line, particularly where affected parties cannot effectively adjust by way of contract.^71^ The result is a neat functional settlement: corporate law and corporate governance take care of profit, while public regulation as a last resort ensures that the profit does not come at public cost. This compartmentalised inside/outside settlement of corporate law originates from the early 20th century, and it still represents the orthodox way of legitimising corporations.^72^

### B. Input Legitimacy of Shareholderism

On the input side, the legitimacy of shareholderism depends on mechanisms or procedures that link corporate decision making with the preferences of relevant stakeholders, ie those affected by the firm’s activities. In the case of shareholderism, the ‘relevant stakeholders’ comprise principally—but not exclusively—shareholders. The main agency costs arise between shareholders and management, and they are mitigated by two types of legal strategies: rights to appoint and remove board members (appointment rights) and rights to decide on certain fundamental matters such as charter amendments, mergers, major transactions and executive compensation (decision rights).^73^ As already mentioned, shareholders are also the sole beneficiaries of directors’ fiduciary duties. The prevalence of these rights across jurisdictions implies that they derive from some normative ideal of ‘shareholder democracy’, which generally refers to the ability of shareholders to use their control rights to influence the corporation.^74^ Such notions of corporate legitimacy influenced the original position of shareholderism, as articulated by Adolf Berle.^75^

The agency cost theory of the firm abandoned shareholder democracy as a normative ideal. Rights are allocated to shareholders not because of some normative ideal of corporate ‘ownership’, but because this solution is the most efficient.^76^ For instance, while Bebchuck argues for legal reforms that would enable shareholders to more directly influence corporate direction, he also notes that he does not view shareholder power ‘as an end in and of itself’.^77^ Giving shareholders intrinsic, independent value could even have value-destructive consequences because the same strategy that allows shareholders to effectively manage their risks, namely the ability to diversify their wealth across several firms, also makes them poor candidates for controlling management.^78^ The result is that shareholders may not have the necessary resources or incentives to vote, monitor management or bring derivative suits against malfeasant directors. Insofar as shareholders only care about profit, as the assumption goes, management is more effectively disciplined by other mechanisms, such as labour and capital markets.^79^ When market mechanisms fail, fiduciary duties and liability rules provide residual safeguards.^80^ The presence of controlling shareholders can also be detrimental for efficiency. Depending on factors such as the underlying market structure, optimal corporate governance may require the population of corporate boards with independent directors that insulate decision making from the demands of controlling shareholders.

The sole purpose of ‘good corporate governance’ is therefore to mitigate agency costs in order to maximise profits. Questions such as what type of powers and how much power should be given to, or retained by, shareholders are relevant only to the extent that they affect output (efficiency). Based on this, the contractarian theory of the firm appears to make no meaningful distinction between input and output. There is only one preference that matters: the profit-interest of shareholders *as a class*. If corporate decision makers maximise profit, they fulfil the requirements of both input and output legitimacy; if they diverge, both suffer. Fretting over the ‘illegitimacy’ of inefficient corporations is useless because the natural forces of competition will make them extinct in any case.

The contractarian view nevertheless ignores the preferences of many stakeholders.^81^ For input legitimacy, the most problematic stakeholders are those that have no contractual relationship with the corporation. As noted by Luca Enriques and others, ‘there is no doubt that the corporation’s economic relevance and impact go well beyond its relationships with contractual counterparties’.^82^ Acknowledging this, Jean Tirole has argued that the realm of corporate governance should be broadened to include *all affected preferences* and defined accordingly ‘as the design of institutions that induce or force management to internalize the welfare of stakeholders’.^83^ Such a broad definition suggests that corporate governance cannot be examined in isolation from the broader legal system, which filters and intermediates stakeholder preferences in shaping corporate conduct through regulatory rules. However, rules alone do not make stakeholders direct beneficiaries of corporate law fiduciary duties, nor do rules, in themselves, compel directors to ‘internalise’ stakeholder preferences.^84^ Rules merely change corporate conduct by their price effects, by making management behave ‘*as if it had the interests of others at heart*’.^85^

### C. The Derivative Legitimacy of the Business Corporation

The main challenge of shareholderism is that the ‘first best’ conditions under which it delivers its promise to maximise social welfare—the absence of externalities and monopolies—are not met.^86^ We therefore have no way of knowing whether the efficiency gains of the corporate system exceed the aggregate costs that it imposes on the planet and its present and future inhabitants.^87^ This poses a serious challenge to the economic justification of shareholderism, both as a normative ideal and as a legal strategy.^88^ For shareholderists, however, these are legitimacy issues that lie with the government, not with the corporation. As noted by Hayek, externalities represent ‘special difficulties’ which the law should remedy through gradual improvement, but they have no connection to the corporate objective problem.^89^ In like terms, Jensen’s defence of shareholder value notes that the resolution of externality and monopoly problems ‘is the legitimate domain of the government in its rule-setting function’.^90^ Shareholderism seldom goes further than this.^91^

This internal/external or private/public domain settlement mirrors certain foundational and underexamined discipline commitments. As is often pointed out, the categoric separation between efficiency and distribution corresponds with the first theorem of welfare economics.^92^ The separation is analytically useful, not least because it provides an effective way to depoliticise the corporate objective discourse. As Duncan Kennedy puts it, it serves to legitimate ‘a virtually exclusive analytical preoccupation with allocation’ while presenting only ‘an implicit appeal to the legislature’.^93^ Corporate law is not the only area of law with such a settlement: bankruptcy and accident law are other examples.^94^

More recently, however, shareholderists’ appeals to the legislature have become more explicit and elaborate. For example, in their defence of shareholderism, Bebchuck and Tallarita list several government interventions—income and carbon taxes, minimum wage regulation, antitrust enforcement, environmental regulations and state subsidies—that could further the interests of corporate stakeholders.^95^ Not all shareholderists would agree with their regulatory prescriptions.^96^ But the government, in its rule-setting function, is increasingly becoming an integral part of the normative framework of shareholderism.^97^ This position approaches the enlightened position of the iconic Adolf Berle, who remained a staunch shareholderist only subject to the proviso that the political process could define the social welfare function and effectively impose it on corporations.^98^

This trend does not, however, question the basic position of shareholderism: corporations should maximise firm value in conformity with the basic rules of the society. If their behaviour does not translate into social welfare, it is because the political process is not holding its end of the ‘social bargain’.

This blame shifting can be criticised from several perspectives. First, as often noted, the capacity of regulators to fix regulatory externalities is limited.^99^ The rationality of regulators is bounded, and the laws are chronically incomplete.^100^ Second, the analytical separation of corporate conduct from politics is unrealistic. Large corporations not only surpass many governments in annual revenue; they also use their resources to influence the political process, and thereby shape the very rules intended to protect stakeholder interests and define the residual sphere of corporate liberty.^101^ Yet, when articulating their appeal to the legislature, shareholderists either relax or ignore the rigorous behavioural assumptions of neoclassical economics. The basic postulate of modern economic theory of regulation (or capture theory) is that we should not expect people to behave less self-interestedly as politicians or bureaucrats than they do as participants in market exchange.^102^ The derivative strategy to legitimate corporations through government regulation is unlikely to be effective without structural reforms insulating policy making and regulation from the excesses of profit-motivated corporate voice.^103^ The absence of corporate political power has with good reason been identified as an additional condition for the normative model of shareholderism.^104^

The third problem concerns the discrepancy between global capital and territorial jurisdiction. Even if a perfectly informed and sanguine political process existed to mitigate the social costs of corporations, the rules may fall short because, in a world of relatively free movement of capital, the laws that apply to multinational corporations are often the laws of their choice.^105^ As already argued by Adam Smith, ‘it is in great measure indifferent to [a merchant] from what place he carries on his trade; and a ‘trifling disgust’ will make him remove his capital, and, together with it, all the industry which it supports, from one country to another’.^106^ No critique of shareholderism fails to raise this problem. The geographic mismatch between capital and jurisdiction has accordingly provided one of the main motivations for calls to re-evaluate the conditions of corporate legitimacy.^107^

Finally, shareholderism can be challenged from a narrower input-legitimacy perspective. Hart and Zingales have argued—in line with the basic demands of input legitimacy—that corporate decision making should be consistent with ‘shareholder welfare’, which is made of genuine investor preferences.^108^ Hart and Zingales are not the first to question the objectification of shareholder value as a form of idealised consensus.^109^ Extensive corporate law scholarship evaluates the diversity of investor preferences and their effect on managerial decision making.^110^ What makes the Hart–Zingales shareholder welfare argument particularly interesting, however, is that it includes no appeal whatsoever to the regulatory functions of government. On the contrary, they argue that corporations with more empowered shareholders could provide a ‘reasonable substitute’ for the ineffective political process.^111^ From the perspective of input legitimacy, this recipe is inadequate. For corporations to act as reasonable substitutes for political processes, shareholder interests should reflect the aggregate interests of societies. This, as many have argued, is not the case.^112^

## 4. Stakeholderism

This section turns to stakeholderism, which includes a thicket of stakeholder-oriented theories from fields such as law, management, business ethics, economics and finance. Compared to shareholderism, a concise and fair presentation of stakeholderism is challenging because it is impossible to point out a dominant theory among them. The approaches are joined by their opposition to shareholder primacy, which they challenge on descriptive, instrumental or normative grounds.^113^ The focus here will be on the normative strand, which adopts the broad principle that corporate decision makers should consider stakeholders as autonomous ends deserving of independent consideration.^114^ In the literature, such approaches have also been labelled ‘pluralistic’^115^ or ‘multi-fiduciary.^116^

As in the preceding section, I will first consider output-oriented justifications before evaluating the importance of input legitimacy in stakeholder theories. The section finishes with a critical evaluation of stakeholderism against the input/output legitimacy criteria.

### A. Output Legitimacy of Stakeholderism

The normative foundations of pluralistic stakeholder theories are versatile and often ambiguous, particularly with respect to output-oriented arguments for legitimacy. Three types of approach will be discussed below: (i) efficiency-oriented approaches; (ii) approaches that mix efficiency explanations with ethical elements; and (iii) normative approaches that subscribe to various conceptions of social value other than efficiency or wealth maximisation.

Efficiency-based stakeholder theories challenge shareholderism on its own terms, claiming that a pluralistic stakeholder approach is the best way to maximise the value of the firm as well as social value. The main premise is that shareholderism fails to account for costs that corporations externalise on stakeholders.^117^ Unlike ‘instrumentalist’ stakeholder theories, which equate corporate performance with profitability, pluralistic approaches assess performance holistically through satisfaction of all relevant stakeholder interests. In such models, profits may be sacrificed to maximise total firm value and social value.^118^

Efficiency-based stakeholder approaches have mainly struggled with issues related to measurement and accountability. As Tirole notes, ‘it is harder to measure the firm’s contribution to the welfare of employees, of suppliers, or of customers than to measure its profitability’.^119^ Without a transparent metric and comparable values, holding management accountable is difficult. More fundamentally, without monetised measures, comparisons would have to made between interpersonal utilities, which is antithetical for efficiency analysis. These problems are overcome if stakeholder interests would have market value just as shares in public companies. Thus, in a recent work, Michael Magill, Martine Quinzii and Jean-Charles Rochet have shown formally how shareholder value maximisation leads to underinvestment compared to stakeholder theory. In their Coasean model, corporate leaders maximise the total value of the firm, an integrated measure comprising share value but also the value of ‘employee rights’ and ‘consumer rights’ that are tradable on a liquid market.^120^ This measure also provides an ‘objective yardstick to judge the performance of the firm’s management’.^121^

In another efficiency-oriented approach, Dirk Schoenmaker, Willem Schramade and Jaap Winter introduce an ‘integrated value measure’ that allows managers to balance financial, social and ecological values. To determine appropriate values, they monetise multiple stakeholder value components using an accounting method known as impact valuation.^122^ This integrated measure, determined through a netting exercise with weights, would be concrete enough ‘to hold managers ex post accountable for their decisions’.^123^

The second category of stakeholder approaches mixes efficiency-based justifications with ethical considerations. In Blair and Stout’s Team Production model, the corporate board, acting as a neutral ‘mediating hierarch’, is responsible for allocating the surplus value among relevant team members. This, they state, facilitates firm-specific investments and maximises the team members’ mutual gain, defined as ‘the joint welfare function of the firm’s members’.^124^ They admit that multi-stakeholder duties would give rise to agency costs, but argue that their theory provides a superior way to mitigate contracting costs. Accordingly, Blair and Stout view their model as compatible with the ‘nexus of contracts’ approach of shareholderism.^125^ Nevertheless, they depart from it by relying on social norms such as integrity and trustworthiness to explain managerial behaviour that would appear irrational in the pure agency cost context.^126^

Mayer’s ‘economics of purpose’ also belongs to the ‘efficiency plus ethics’ family of stakeholder-oriented approaches. He suggests that stakeholder capitalism would enhance the productive potential of societies by relieving ‘constraints on productive capabilities’.^127^ Emphasising the problem-solving capacity of corporations, Mayer’s approach draws primarily from output legitimacy. For him, corporations are vehicles uniting all to ‘a common goal of producing profitable solutions to problems of people and planet’.^128^ This could be achieved by redefining ownership in a way that makes companies internalise externalities.^129^ Here, Mayer does not disagree with mainstream economics.^130^ What he challenges is the established wisdom of welfare economics, which leaves issues of distribution to governments.^131^ Instead, concerns such as fairer opportunities should become part of the very problems that corporations exist to solve.^132^ Mayer’s approach finds its ultimate normative basis in a specific theory of property: shareholders as the owners of corporations have special responsibilities towards external parties.^133^ This *moral obligation* also explains why, according to Mayer, shareholder profit should never be earned at the expense of other parties, but profits can be sacrificed for the benefit of other stakeholders.^134^

The third category of stakeholder approaches, which is most varied, need not be examined in detail here. It includes normative theories grounded on ethical theories or axiomatic principles that are less concerned with quantifiable notions of social welfare. For instance, in their neo-utilitarian model, Thomas Jones and Will Felps equate social welfare with ‘aggregate happiness’ of the corporation’s legitimate stakeholders.^135^ Ronald Mitchell and others also advocate a broad conception of social welfare that includes ‘sacred’ and non-negotiable goods such as life satisfaction and meaningfulness.^136^

Finally, ‘corporate sustainability’ should be singled out as a specific approach that is particularly difficult to categorise in terms of output legitimacy. It is more concerned with minimising harm than with maximising some measure of social value, admitting that there is no such thing as a ‘sustainability maximand’ that could replace shareholder primacy.^137^ Instead, the natural sciences concept of ‘planetary boundaries’ should set the outer limits beyond which no economic activity should go. Accordingly, corporate sustainability would involve ‘corporate legal and governance structures promoting practices that contribute to and, at a minimum, do not undermine society’s potential for achieving the overarching goal of sustainability’.^138^

### B. Input Legitimacy of Stakeholderism

As with shareholderism, the legitimacy of stakeholderism on the input side depends on mechanisms and procedures that ensure corporate decision makers engage with stakeholders’ goals and preferences, and remain accountable to them. The most straightforward way to enable stakeholders to voice their preferences would be to empower them, like shareholders, to elect and remove directors, enforce fiduciary duties, vote on major transactions and so on. It has even been argued that stakeholderism cannot be taken seriously before shareholders’ powers are shared with stakeholders.^139^ The above-described economic model of Magill, Quinzii and Rochet acknowledges this: to ensure accountability, the tradable rights of consumers and workers are also attached with legal voting rights.^140^

Nevertheless, many influential stakeholder theories have downplayed the desirability of such formal power-sharing arrangements. In Blair and Stout’s team production model, *no stakeholder*, including shareholders, has direct control over the board.^141^ The board need not even be informed of stakeholder interests. The job of the mediating hierarch is to ‘keep everyone happy enough that the productive coalition stays together’,^142^ and it achieves this for the most part by being *uninformed of and passive about* stakeholder conflicts. Where disputes arise among team members, they should primarily be worked out among themselves. The rationale here is that the mediated result is likely to be more acceptable than the alternative of ‘kicking the problem upstairs to a disinterested, but potentially erratic or ill-informed, hierarch’.^143^

The corporate purpose scholarship is similarly wary of giving formal control powers to stakeholders. Mayer argues that this would come ‘at the expense of the party which is not represented at all—future generations’.^144^ Managerial incentives should not be aligned with the haphazard and selfish interests of stakeholders, but with the purpose of the corporation as established by the board itself. Corporate purpose would be firm-specific, but it would have to respect the general legal maxim ‘to provide profitable solutions to the problems of people and planet, and not profit from producing problems for either’.^145^ While it is acknowledged that setting the purpose necessarily involves making judgments about stakeholders’ interests, little is said about the role of stakeholders in formulating it.^146^ However, corporations could establish ‘councils, forums and supervisory boards’ to foster inclusive accountability.^147^ Diverse board structures would also help establish responsiveness to a range of stakeholders, but no guidance is given as to how this could be achieved.^148^ As regards legal control, the only stakeholder emphasised is the long-term blockholder, who should be appropriately empowered.^149^

Similar conclusions have been reached in the corporate sustainability literature. For example, Beate Sjåfjell and Jukka Mähönen argue that global challenges ‘will not be resolved through identifying possible stakeholders and their private preferences’.^150^ Replacing shareholder primacy with ‘stakeholder primacy’ would only increase the voice of capital providers at the expense of the under-represented voices of future generations and the environment. Instead, science-based concepts of ‘sustainable value’ and ‘planetary boundaries’ should be recognised as general clauses in company law and be incorporated into corporate purpose.^151^ They also suggest that these general concepts could be integrated into the fiduciary duties of the board, but provide little analysis on how, and by whom, such sustainability-oriented directors’ duties would be enforced, beyond ensuring that decision making complies with certain procedural requirements.^152^

Kershaw and Schuster have also argued that the ‘purposive transformation’ of corporate law requires that managers enjoy a ‘zone of insulation’ from non-aligned shareholder pressure. Their approach, however, emphasises legal optionality and—like shareholderists –views corporate law ideally as an ‘adaptive toolbox for a diverse range of companies’.^153^ They do not argue for making boards wholly unaccountable to shareholders, only that they should be less receptive to shareholders’ immediate demands.^154^

Some ‘contractarian’ stakeholder approaches take stakeholder preferences more seriously. The formal stakeholder theory of Magill, Quinzii and Rochet, discussed earlier in this section, provides a recent example.^155^ Non-formal theories in management studies have also attempted to update the predominant agency theory view of the firm with a broader range of stakeholder interests.^156^ In terms of input legitimacy, such approaches are as complete as the ‘markets’ where stakeholder preferences are signalled. Recent legal literature has also given more weight to stakeholder preferences.^157^ The ‘new model of corporate governance’ introduced by Grant Hayden and Matthew Bodie is particularly interesting, as it draws from the theory of democratic participation in emphasising the importance of stakeholder preferences and rights.^158^

These ‘representative models’ of stakeholder theory remain in the minority.^159^ The majority of existing approaches, including contemporary advocates of corporate purpose and corporate sustainability, retain the appointment monopoly of shareholders while granting directors greater discretion to consider the interests of other stakeholders.^160^

### C. Searching for ‘the Legal Version of Stakeholder Theory’

The only theme uniting the expanding gallery of stakeholder approaches is their adversary: shareholder primacy. Some scholars considered here as ‘stakeholderist’ have also resisted the label and argued for abandoning the shareholderism/stakeholderism dichotomy entirely.^161^ However, each of the above-considered normative approaches can be broadly interpreted as subscribing to the basic position according to which corporate decision makers should consider stakeholders ‘as ends in themselves’, not as instruments to achieve some other goal. This idea still forms the normative core of (pluralistic) stakeholderism.^162^

Against this unifying principle, much of the criticism of stakeholderism is captured by the following remark by Bertrand Russel:

If each is an end in himself, how are we to arrive at a principle for determining which shall give way? Such a principle must have to do with the community rather than with the individual. In the broadest sense of the word, it will have to be a principle of ‘justice’.^163^

Russell’s quote entails two related elements, both of which resonate with the input- and output-related demands of legitimacy: first, conflict resolution must be based on a concrete normative principle; and second, that principle must correspond with some idea of ‘community’ and be responsive to its interests. How does stakeholderism respond to these demands?

To start with output legitimacy, how should corporate leaders weigh and prioritise among competing needs in the inevitable situation that decisions must be made that benefit some stakeholders more than others, or even benefit one stakeholder at the cost of another? For example, by which calculus or set of principles would leaders of a multinational corporate conglomerate balance ecological values, such climate change or biodiversity, with human values, such as diminishing poverty and hunger, all of which are United Nations sustainable development goals? The stakeholderists’ inability to answer this question convincingly has been considered one of its main weaknesses. It was standard criticism against the Corporate Social Responsibility movement^164^ and often raised by the contemporary critics of stakeholderism as well.^165^

In closer scrutiny, however, the problem is not the lack of such normative principles so much as their plurality. Suggested normative bases for stakeholderism include, at least, Rawlsian, Kantian, ecological, utilitarian, neo-utilitarian, libertarian, feminist as well as social contract theory and property theory.^166^ Such diversity also explains the pervasive disagreement on who to include in the ‘corporate community’ of stakeholders. Whether one seeks to maximise the ‘sum of the stakeholders’ surpluses’,^167^ the sum of stakeholders’ happiness^168^ or some other metric, one must first establish *whose* surplus, happiness and so on counts towards the final figure. Stakeholderists—short of a unifying ‘normative theory of stakeholder identification’—do not agree on this.^169^ It is not even clear whether the criteria for stakeholder identification should be objective, as in Freeman’s often-cited definition,^170^ or subjective, determined by corporate leaders or stakeholders themselves.^171^

For many contemporary stakeholderists, the responsibility to make these choices falls to corporate boards with responsibility to define and implement the corporate purpose. Corporate purpose established by the board could provide a ‘guiding star’ which helps resolve trade-offs between different stakeholders.^172^ Corporate sustainability scholarship agrees, but adds ‘sustainable value’ as a dynamically evolving scientific concept that sets outer limits to managerial discretion. Efficiency-oriented stakeholder models also leave discretion to corporate leadership to determine ‘the weights’ given to relevant stakeholders in corporate decision making.^173^

Such managerialist conflict resolution raises two legitimacy-related issues. First, to what extent are directors *informed of and responsive to* the interests of relevant stakeholders when establishing the purpose of the corporation? This input legitimacy issue will be considered in the next subsection. The remainder of this subsection focuses on the second issue of accountability: how to ensure the fidelity of managers to whatever purpose they have committed to follow?

An explicit identification of a corporate purpose does not alone render an objective and enforceable standard of managerial conduct. Corporate purpose clauses—at least in their current inconcrete and unenforceable form—do not provide a credible constraint against managerial opportunism.^174^ Thus, shareholderists argue, leaders of a corporation could rationalise any action taken by invoking the affected welfare of some stakeholder.^175^ Consequently, corporate leaders are left free ‘to exercise their *own preferences* in spending the firm’s resources’.^176^ This is a serious argument against stakeholderism, challenging its legitimacy in both input- and output-related terms. The preferences of corporate leaders are not necessarily the same as those who are affected by their decisions (input). And by expanding the powers of self-interested managers, stakeholderism reduces ‘the economic pie’ available for everyone (output). This is not only because of increased agency costs, but also because stakeholderism would deflect attention from more effective means (regulation) to address legitimate stakeholder concerns.^177^

Stakeholderists have countered this scepticism by designing versatile schemes for operationalising stakeholderism. As noted above, efficiency-oriented models have addressed the problem by means that are conceptually compatible with shareholderism.^178^ For example, an ‘integrated value’ combining monetised measures of financial, human, social, intellectual and natural capital would, according to Schoenmaker, Schramade and Winter, solve the efficiency problem of ‘multiple goals or masters’.^179^ The board’s accountability would further be ensured by a mixture of governance arrangements, such as a stakeholder council (the board of which would meet once a year and consult where necessary), sustainability-sensitive executive compensation and formal board mandates (set by directors themselves) geared towards sustainability.^180^ In this model, stakeholders would not need formal voting power. The only problem that remains is that the necessary technologies and scientific knowledge to derive sufficiently robust quantitative values do not yet exist.^181^ And even if they did, not everything, such as the interest of future generations, can be measured and monetised.^182^

Other approaches acknowledge the need for effective accountability but defer solutions to future research. Donaldson and Preston, for example, suggest that their stakeholder model would effectively curb managerial opportunism, if only it was supported by the necessary laws, sanctions, rules and precedents. This ‘legal version of the stakeholder model’ did not yet exist, but it would, they predicted, ‘almost certainly’ be achieved.^183^

Corporate purpose literature also highlights the need for robust accountability structures ensuring that corporations deliver on their established purposes.^184^ To that end, the British Academy’s Future of the Corporation project has identified several high-level principles guiding reforms in legal, regulatory, governance and reporting frameworks. It is doubtful whether the ‘legal version of the stakeholder model’ can be built on these aspirational principles. The principles do not suggest giving direct control rights to non-shareholder groups or broadening the scope of court-enforced fiduciary duties of corporate directors.^185^ As the final report of the project indicates, reforming business is not just about laws and regulations; it is also about values and culture that foster commitment and cooperation.^186^

The accountability problem can be elegantly circumvented by arguing that the entire problem is wrongly framed. Some stakeholder approaches accordingly suggest that managerial opportunism—where it exists—is due to decision makers’ adherence to the misplaced *social norm* of shareholder primacy. Liberated from this morally destructive norm or ideology, and the short-termism of markets and activist investors, corporate directors could use the discretion already afforded to them by law to uphold the well-being of others.^187^ Corporate leaders would thus need to be policed less by externally enforced legal standards or markets and more by ‘responsible self-policing’,^188^ social and moral sanctions such as reprobation and feelings of guilt,^189^ or a desire for prestige and other non-pecuniary rewards.^190^

In normative terms of output legitimacy, the shareholderism versus stakeholderism debate eludes a neat resolution. No analytical framework exists to decisively determine whether the social costs of shareholderism outweigh the agency costs associated with stakeholderism. The debate, as suggested before, is inconclusive.^191^ However, the problems of stakeholderism become more apparent in the participatory dimension of input legitimacy. Whatever concept of social value a stakeholder approach subscribes to, that measure should—at least based on the definition of input legitimacy adopted here—be responsive to the individual ends that the relevant corporate constituents care about. As the next subsection shows, stakeholderism has sought to address these demands via innovative, but ultimately unconvincing, mechanisms.

### D. A Deliberative Model of Corporate Governance?

As the preceding subsection showed, many stakeholderists would substitute an open-ended set of company-specific objectives for the universal default purpose of shareholder value. This, of course, is an exercise in normative judgment:

The issue of the overarching purpose of the company of course is essentially a normative question. It must be determined based on a view of what the company is meant to do, on which interests are involved or affected and on how those various interests combine and relate to the common good.^192^

For Mayer, the variety of corporate purposes is limited only by the requirement that they align with ‘common goals’ of people and planet.^193^ Given such particularism, stakeholder theories appear to flirt with the political philosophies of republicanism or communitarianism, which are primarily concerned with the orientation of polities, prioritised over individuals, towards different notions of ‘common good’.^194^ Few stakeholderists would welcome such philosophical associations. If the republican tradition evolved to adopt democratic representation, electoral accountability and universal suffrage as key constitutional mechanisms to ensure the fidelity of governors to ‘common good’,^195^ stakeholder theories have moved in the opposite direction: first becoming agnostic about the value of stakeholder democracy (ie giving stakeholders voting rights and other forms of formal control over corporations)^196^ and now increasingly rejecting it. In fact, even self-labelled communitarians within corporate legal theory have sought to shrug off any ‘antiliberal connotations’.^197^

But it is hard to place the interests of individual stakeholders in all of this. If individual preferences are to serve as inputs to a process of social choice, which the determination of corporate purpose necessarily entails, they would have to be expressed, not taken as given.^198^ How, then, does stakeholderism seek to (input) legitimise corporations in a system of governance in which the increasing political authority of corporations coincides with gradually eroding state power?^199^

To tackle this problem, some scholars have turned to public law as a source of inspiration. This move is both predictable and sensible, as critics of shareholderism often portray corporations as public bodies using public power.^200^ If this view is accepted, the conceptual and practical challenges of corporate accountability become analogous to those involved in integrating independent agencies into a system of accountable government.^201^ In their ‘public-law blueprint for corporate governance’, Gadinis and Havasy accordingly explore the toolkit of administrative law to promote goals such as transparency, accountability, consultation and reason giving in corporate decision making, which they see as neglected means to enhance corporate legitimacy.^202^

Such ideas have also been expressed in recent legislation. In terms of *participation* and *engagement*, the prime example is the European Union’s Corporate Sustainability Due Diligence Directive (EU CSDDD), which obliges large corporations to engage effectively with stakeholders.^203^ As the Directive explains, effective engagement should entail ‘providing consulted stakeholders with relevant and comprehensive information, as well as ongoing consultation that allows for genuine interaction and dialogue at the appropriate level’.^204^ The EU Corporate Sustainability Reporting Directive^205^ fosters *transparency* by requiring large corporations to disclose information in relation to various social and environmental issues, and on their impact on people and the environment. The Directive responds to the information needs of sustainable financial markets but also to the needs of stakeholders, who need sustainability-related information to hold corporations ‘accountable for their impacts on people and the environment’ and ‘to enter into dialogue’ with them.^206^

Full exploration of the legitimacy challenges of the administrative law approach to corporate legitimation is beyond the reach of this article. To be compatible with the liberal-democratic model of delegation, however, several requirements would have to be met. For example, to ensure political accountability, the mandates and objectives of regulatory agencies must always be statute-defined.^207^ The procedural requirements advocated by some stakeholderists—as well as those set out in the EU CSDDD—are nevertheless concerned with the *implementation* of corporate purposes and board mandates rather than their establishment. There is little support, even among contemporary stakeholderists, for returning to a concession system where corporations and their objectives were authorised by parliamentary acts.^208^ Second, the exercise of powers by non-majoritarian institutions must generally be amenable to effective judicial review also based on their outcomes. This is not the case for corporations. Without a legally binding mandate limiting corporations to specific purposes and powers, a legality review by a court can at best consider whether corporate conduct is otherwise lawful.^209^ Updating the process-oriented business judgment rule to incorporate a form of ‘social judgment’ would hardly help either, unless the judicial deference to outcomes that is inherent in the business judgment rule were abandoned.^210^ Finally, there are generally limits to the type of power that can be delegated to technocratic institutions using public power. The basic requirement is that redistributive policies should never be delegated to institutions independent of the political process; the legitimation of such powers requires majoritarian means involving the electorate.^211^

These legitimacy challenges diminish considerably if one abandons the liberal-democratic frame. In a *deliberative model of democracy*, the inputs to social choices are not made of the raw and selfish individual preferences that operate in the marketplace, but of other-regarding preferences as shaped in public debates geared towards common good.^212^ As Elster notes, the goal of politics on this latter view is ‘not optimal compromise, but unanimous agreement’.^213^ Stakeholder-oriented deliberation, supported by mandatory rules fostering transparency and consultation, could therefore itself serve as a standard of legitimacy.

This consensus-seeking deliberative model, deriving from Jürgen Habermas, has been wholeheartedly embraced by Scherer and Palazzo in the context of corporate governance.^214^ For them, globalised corporations can gain legitimacy primarily through justifying their actions in input processes of civic engagement and interaction, in which private preferences are not only expressed, but possibly also transformed.^215^ Similar approaches have been advocated in governance studies to remedy participatory deficits and the resulting legitimacy gaps in global governance.^216^

The promises and pitfalls of the deliberative model of democratic legitimation have been extensively debated by political scientists.^217^ Against the input/output criteria of legitimacy adopted here, the strategy appears dangerously inadequate. As Julia Black has argued, the deliberative model is both complex to develop and challenging to defend.^218^ To work, it would have to compensate ‘for the distorting effects of power relationships that are both internal and external to the deliberation’.^219^ One may question whether the proposed models of ‘deliberative corporate governance’ have succeeded in identifying, let alone compensating for, the distorting effects of such power imbalances. The deliberative model is primarily concerned with procedural values, focusing on *how* certain social ends can be achieved, while diverting attention from the question of *who* should define those ends.^220^ This applies to the EU CSDDD as well, which contains ‘obligations of means’, not ends.^221^

By leaving substantive value choices to self-authorised corporations operating outside comprehensive accountability frameworks, the deliberative turn in corporate governance is bound to attract criticism similar to that faced by the Global Administrative Law project: it reinforces and stabilises existing power structures and inequalities, rather than offering tools for meaningful empowerment and change.^222^ Depending on how corporate managers, based on their self-defined ‘guiding stars’, weigh different stakeholder interests, ‘some people … will benefit, while others will face deepening deprivation’.^223^

## 5. Conclusions

More than 40 years ago David Engel and Roberta Romano criticised Corporate Social Responsibility reformists for presenting proposals without sufficient normative reflection, for focusing too much on means or reform while failing ‘to articulate the vision of the good society’ that informed their policy programmes.^224^ This observation remains accurate. The heterogeneous group of stakeholder theories does not present an opposing force joined by a coherent system of thought. As many social scientists,^225^ they accept that globalisation has severely challenged the regulatory capacities of governments, and indeed their legitimacy. Yet, the principal strategy of stakeholderism to deal with corporate power appears to be co-option rather than containment. It is not clear whether this is because corporate power and free movement of capital are accepted as undesirable structural conditions that are more difficult to change than existing systems of corporate law and governance or because they are essential elements of some unidentified corporatist or other political ideal in which the power of corporations is not reduced so much as made to serve the ‘public good’. In the latter case, it will be corporations, appropriately reformed to internalise their public-spirited duties, that can restore not only corporate legitimacy, but also the legitimacy of governments.

The political visions of shareholderism, on the other hand, remain for the most part unchanged: it compartmentalises issues of efficiency (corporate system) and distribution (political system) into different boxes with different means of legitimation. This respects the fundamental requirement of the liberal-democratic theory of delegation: only the majoritarian means of voting and electoral representation can legitimise redistributive policies, which, transformed into effective rules, should legitimise the residual corporate power. These may not make corporate management internalise stakeholder interests in its decision making, but appropriate rules at least make it behave ‘as if it had the interests of others in mind’.^226^

But if stakeholderism is ambiguous about its ultimate ends, the problem of shareholderism is that it pays insufficient attention to *means*: despite deriving its continuing legitimacy from governments (in both input and output dimensions), shareholderists fail to address the structural reasons for governments’ persistent failure to address corporate externalities.

In terms of output legitimacy, the shareholderism versus stakeholderism debate remains inconclusive. We do not know whether it is more effective to fix corporate externalities and monopoly problems via taxes, regulation or other forms of direct government action (shareholderism) than via some elaborate scheme of rules, practices, social norms and values that incentivise corporate leaders to behave pro-socially by using their own best judgment (stakeholderism). This disagreement can only be settled by empirical evidence, which is patchy and biased.^227^ The alternative of stakeholderism is therefore at least plausible. In terms of input legitimacy, however, stakeholderism clearly falls short. The emerging proceduralist agenda of transforming corporations into deliberative and distributive polities, governed by principles such as transparency and communication, offers but a thin basis for corporate legitimacy.

## Footnotes

1 The literature on the topic is vast and growing, but the exchange in Cornell Law Review between Lucian A Bebchuk and Roberto Tallarita on the one hand and Colin Mayer on the other represents the zenith of the contemporary debate. See Lucian A Bebchuk and Roberto Tallarita, ‘The Illusory Promise of Stakeholder Governance’ (2020) 106 Cornell L Rev 91; Colin Mayer, ‘Shareholderism versus Stakeholderism—A Misconceived Contradiction: A Comment on “The Illusory Promise of Stakeholder Governance” by Lucian Bebchuk and Roberto Tallarita’ (2021) 106 Cornell L Rev 1859. The historical antecedents were laid in the 1930s in the more famous exchange between Adolf Berle and Merrick Dodd. See Adolf A Berle Jr, ‘Corporate Powers as Powers in Trust’ (1931) 44 Harv L Rev 1049; Adolf A Berle Jr, ‘For Whom Corporate Managers Are Trustees: A Note’ (1932) 45 Harv L Rev 1365; E Merrick Dodd Jr, ‘For Whom Are Corporate Managers Trustees?’ (1932) 45 Harv L Rev 1145. For a historical overview of the great corporate purpose debate and predictions of its future trajectories, see Brian C Cheffins, ‘The Past, Present and Future of Corporate Purpose’ (2024) 48 Del J Corp L 387.

2 See The British Academy, *Policy & Practice for Purposeful Business. The Final Report of the Future of the Corporation Programme* (2021), noting that the ‘[sterile] debate of shareholder versus stakeholder has dominated discussion of company law for decades, without much advancement’.

3 David L Engel, ‘An Approach to Corporate Social Responsibility’ (1979) 32 Stan L Rev 1, 1.

4 Roberta Romano, ‘Metapolitics and Corporate Law Reform’ (1984) 36 Stan L Rev 923, 924.

5 ibid 924; Engel (n 3) 4.

6 Stavros Gadinis and Chris Havasy, ‘The Quest for Legitimacy: A Public Law Blueprint for Corporate Governance’ (2024) 57 UC Davis L Rev 1581, 1593, 1598–1604.

7 Bebchuk and Tallarita (n 1) 168.

8 Similar justifications for corporate legitimacy-related inquiries have been given in Cary Coglianese, ‘Legitimacy and Corporate Governance’ (2007) 32 Del J Corp L 159, 161; Gadinis and Havasy (n 6) 1598–1604 and Edwin M Epstein, ‘The Historical Enigma of Corporate Legitimacy’ (1972) 60 Calif L Rev 1701, 1709.

9 See Leo E Strine, ‘Restoration: The Role Stakeholder Governance Must Play in Recreating a Fair and Sustainable American Economy: A Reply to Professor Rock’ (2020) 76 Bus Law 76, 397, 421–3; William Magnuson, *For Profit: A History of Corporations* (Basic Books 2022) ch 1.

10 David Beetham, *The Legitimation of Power* (2nd edn, Bloomsbury Publishing 2013) 39. On legitimacy of market transactions, see Fabienne Peter, ‘Choice, Consent, and the Legitimacy of Market Transactions’ (2004) 20 Economics & Philosophy 1.

11 Fritz W Scharpf, *Governing in Europe: Effective and Democratic?* (OUP 1999) 6.

12 Adapted from Fritz W Scharpf, ‘Economic Integration, Democracy and the Welfare State (1997) 4 Journal of European Public Policy 18 19.

13 This, however, being just one of the three legitimating factors of what he calls the ‘Economic Model’ of legitimacy. See Melvin Eisenberg, ‘Corporate Legitimacy, Conduct, and Governance: Two Models of the Corporation’ (1983) 17 Creighton L Rev 1, 5.

14 Milton Friedman, ‘The Social Responsibility of Business Is to Increase Its Profits’ *New York Times Magazine* (13 September 1970) 32.

15 Edward B Rock, ‘For Whom Is the Corporation Managed in 2020? The Debate over Corporate Purpose’ (2021) 76 Bus Law 363, 394. Compare Andreas G Scherer and Guido Palazzo, ‘Toward a Political Conception of Corporate Responsibility: Business and Society seen from a Habermasian Perspective’ (2007) 32 Academy of Management Review 1096, 1107.

16 Henry G Manne, ‘The Higher Criticism of the Modern Corporation’ (1962) 62 Colum L Rev 399, fn 95.

17 John Rawls, *A Theory of Justice* (rev edn, Harvard UP 1999) 126. For further discussion, see Abraham Singer, ‘There Is No Rawlsian Theory of Corporate Governance’ (2015) 25 Business Ethics Quarterly 65.

18 David Millon, ‘Theories of the Corporation’ (1990) Duke LJ 201 202. The extensive debate is summarised well by Alan Wolfe, ‘The Modern Corporation: Private Agent or Public Actor’ (1993) 50 Wash & Lee L Rev 1673, 1675. Whether corporations should be conceived as (private) contractual arrangements or as real entities is another major conceptual debate. For a recent in-depth discussion on the competing approaches, see Eva Micheler, *Company Law: A Real Entity Theory*’ (OUP 2021).

19 William T Allen, ‘Our Schizophrenic Conception of the Business Corporation’ (1992) 14 Cardozo Law Review 261, 281.

20 James J Brummer, *Corporate Responsibility and Legitimacy: An Interdisciplinary Analysis* (Greenwood Press 1991) 80–95.

21 Joseph H Weiler, ‘In the Face of Crisis: Input Legitimacy, Output Legitimacy and the Political Messianism of European Integration’ (2012) 34 Journal of European Integration 825, 826.

22 Beetham (n 10) x.

23 ibid.

24 ibid 4.

25 Gadinis and Havasy (n 6) 1598–9.

26 Ian B Lee, ‘Efficiency and Ethics in the Debate About Shareholder Primacy’ (2006) 31 Del J Corp L 533, 575. Even the ‘Friedman doctrine’, which posits that the sole social responsibility of business is to maximise profits, accepts that this should be done ‘while conforming to the basic rules of the society, both those embodied in law and those embodied in ethical custom’: Friedman (n 14). Identical argument is presented in Friedrich Hayek, ‘The Corporation in a Democratic Society: In Whose Interest Ought It To and Will it Be Run?’ in Friedrich Hayek (ed), Studies in Philosophy, Politics and Economics (first published 1960, Routledge & Kegan Paul 1967) 301.

27 Beetham (n 10) 4. The issue is plainly stated by Weiler (n 21) 827: ‘Forbidding blacks to sit in the front of the bus was perfectly legal, but would fail many a test of normative legitimacy.’

28 Eric W Orts, ‘The Complexity and Legitimacy of Corporate Law’ (1993) 50 Wash & Lee L Rev 1565.

29 Richard A Posner, ‘Utilitarianism, Economics, and Legal Theory’ (1979) 8 JLS 103, 129–30.

30 James W Hurst, *Legitimacy of the Business Corporation in the Law of the United States 1780–1970* (The University Press of Virginia 1970).

31 ibid 58.

32 On the difference between internal and external accountability and how they relate to legitimacy, see Thomas Risse, ‘Transnational Governance and Legitimacy’ in Arthur Benz and Yannis Papadopoulos, *Governance and Democracy* (Routledge 2006) 179–99.

33 For example, if production of wealth is viewed as part of the ‘economic responsibility’ of corporations, behaving responsibly becomes an integral part of maximising utility. See Brummer (n 20) 20. Epstein, reviewing Hurst’s essay, also predicts the disappearance of the distinction between utility and responsibility. See Epstein (n 8) 1715.

34 Jens Steffek, ‘The Democratic Output Legitimacy of International Organizations’ (Wissenschaftszentrum Berlin für Sozialforschung, 2014) WZB Discussion Paper No SP IV 2014-101.

35 Steffek refers to such procedures as ‘epistemic’. See ibid 5–6.

36 ibid 22.

37 See ibid 22; Giandomenico Majone, ‘Europe’s “Democratic Deficit”: The Question of Standards’ (1998) 4 Eur L J 5, 28.

38 As Elster notes, this requirement is a fundamental tenet of social choice theory. See Jon Elster, ‘The Market and The Forum: Three Varieties of Political Theory’ in James Bohman and William Rehg (eds), *Deliberative Democracy: Essays on Reason and Politics* (MIT Press 1997) 6.

39 Beetham (n 10) 267.

40 Fritz W Scharpf, ‘Problem-Solving Effectiveness and Democratic Accountability in the EU’ (2003) MPIfG Working Paper 03/1.

41 David Estlund, ‘Democratic Theory’ in Frank Jackson and Michael Smith (eds), *The Oxford Handbook of Contemporary Philosophy* (OUP 2007) 208.

42 See Gadinis and Havasy (n 6) 1597.

43 Within corporate law scholarship, this genre includes the growing literature on ‘social license’ of corporations to operate. See eg Hillary A Sale, ‘The Corporate Purpose of Social License’ (2021) 94 S Calif L Rev 785.

44 Weiler (n 21) 826.

45 Risse (n 32); Karin Bäckstrand, ‘Multi-Stakeholder Partnerships for Sustainable Development: Rethinking Legitimacy, Accountability and Effectiveness’ (2006) 16 European Environment 290; Sébastien Mena and Guido Palazzo, ‘Input and Output Legitimacy of Multi-Stakeholder Initiatives. (2012) 22 Business Ethics Quarterly 527;

46 As noted by Steffek, ‘few conceptual contributions have left more of an imprint on [the agenda of international political theory and European studies] than the distinction between input and output legitimacy’. Steffek, ‘The Democratic Output’ (n 34) 2.

47 As Scharpf puts it, ‘democracy would be an empty ritual if the political choices of governments were not able to achieve a high degree of effectiveness in achieving the goals, and avoiding the dangers, that citizens collectively care about’. Scharpf, ‘Economic Integration’ (n 12) 19.

48 Steffek, ‘The Democratic Output’ (n 34) 5.

49 ibid 26.

50 Bäckstrand (n 45) 294–5.

51 Jens Steffek, ‘The Limits of Proceduralism: Critical Remarks on the Rise of “Throughput Legitimacy”’ (2019) 97 Public Administration 784, 790.

52 Renée B Adams, Amir N Licht and Lilach Sagiv, ‘Shareholders and Stakeholders: How Do Directors Decide?’ (2011) 32 Strategic Management Journal 1331, 1332.

53 Amir N Licht, ‘Varieties of Shareholderism: Three Views of the Corporate Purpose Cathedral’ in Elizabeth Pollman and Robert B Thompson (eds), *Research Handbook on Corporate Purpose and Personhood* (Edward Elgar Publishing 2021) 389.

54 Frank H Easterbrook and Daniel R Fischel, *The Economic Structure of Corporate Law* (Harvard UP 1996).

55 For a review contending theories, see David Kershaw and Edmund Schuster, ‘The Purposive Transformation of Corporate Law’ (2021) 69 Am J Comp L 478, 520, and the literature referenced therein. See also David Millon, ‘Radical Shareholder Primacy’ (2013) 10 University of St Thomas Law Journal 1013; Stephen M Bainbridge, ‘Director Primacy: The Means and Ends of Corporate Governance’ (2002) 97 Nw U L Rev 547, 563.

56 Michael C Jensen, ‘Value Maximization, Stakeholder Theory, and the Corporate Objective Function’ (2002) Business Ethics Quarterly 235, 239–40.

57 ibid 239.

58 Frank H Easterbrook and Daniel R Fischel, ‘The Corporate Contract’ (1989) 89 Colum L Rev 1416, 1446.

59 Eugene F Fama and Michael C Jensen, ‘Agency Problems and Residual Claims’ (1983) 26 JLE 327, 327. The most influential characterisation of agency costs is presented in Michael C Jensen and William H Meckling, ‘Theory of the Firm: Managerial Behavior, Agency Costs and Ownership Structure (1976) 3 Journal of Financial Economics 305.

60 Jonathan R Macey, ‘An Economic Analysis of the Various Rationales for Making Shareholders the Exclusive Beneficiaries of Corporate Fiduciary Duties’ (1991) 21 Stetson L Rev 23, 25; Oliver Hart, ‘An Economist’s View of Fiduciary Duty’ (1993) 43 UTLJ 299; Jill E Fisch, ‘Measuring Efficiency in Corporate Law: The Role of Shareholder Primacy’ (2005) 31 J Corp L 637.

61 Jensen (n 56) 242. As phrased by Easterbrook and Fischel, ‘a manager told to serve two masters … has been freed of both and is answerable to neither’. Easterbrook and Fischel, *The Economic Structure of Corporate Law* (n 54) 38.

62 Abraham A Singer, *The Form of the Firm: A Normative Political Theory of the Corporation* (OUP 2019) 107–8.

63 Easterbrook and Fischel, *The Economic Structure of Corporate Law* (n 54) 15, 34.

64 John Armour and others, ‘What Is Corporate Law?’ in Reinier Kraakman and others (eds), *The Anatomy of Corporate Law: A Comparative and Functional Approach* (3rd edn, OUP 2017) 23. See also Jensen (n 56) 239, arguing that ‘200 years of worth of work in economics and finance indicate that social welfare is maximized when all firms in an economy maximize total firm value’.

65 Armour and others, ‘What Is Corporate Law?’ (n 64) 22–3.

66 See Jensen (n 56) 239–40; Armour and others, ‘What Is Corporate Law?’ (n 64) 23, 30–1.

67 Jean Tirole, ‘Corporate Governance’ (2001) 69 Econometrica 1, 2.

68 Armour and others, ‘What Is Corporate Law?’ (n 64) 23 fn 87.

69 Jensen (n 56) 239.

70 Mark J Roe, ‘The Shareholder Wealth Maximization Norm and Industrial Organization’ (2001) 149 U Pa L Rev 2063.

71 John Armour and others, ‘Beyond the Anatomy’ in Kraakman and others (n 64) 271.

72 William W Bratton and Michael L Wachter, ‘Shareholder Primacy’s Corporatist Origins: Adolf Berle and the Modern Corporation’ (2008) 34 J Corp L 99, 150. Hurst (n 30) provides a vivid historical account of the evolution of corporate law in the United States towards this inside/outside settlement.

73 See generally John Armour and others, ‘The Basic Governance Structure: The Interests of Shareholders as a Class’ in Kraakman and others (n 64).

74 Anita I Anand, *Shareholder-Driven Corporate Governance* (OUP 2021) ch 3.

75 For Berle, shareholder vote was the main standard for the legitimacy of management rule and corporate power. If shareholder support was lacking or merely nominal, then corporate power required other means of legitimation. For discussion, see Singer (n 62) 82.

76 Easterbrook and Fischel, ‘The Corporate Contract’ (n 58) 68–9.

77 Lucian A Bebchuck, ‘The Case for Increasing Shareholder Power’ (2004) 118 Harv L Rev 833, 842.

78 Eugene F Fama, ‘Agency Problems and the Theory of the Firm’ (1980) 88 J Polit Econ 288

79 ibid; Easterbrook and Fischel, ‘The Corporate Contract’ (n 58) 98.

80 Easterbrook and Fischel, ‘The Corporate Contract’ (n 58) 103.

81 Some shareholderist accounts consider that the remit of corporate law may extend to certain non-shareholder constituencies, particularly creditors, but possibly also workers. See generally Kraakman and others (n 64) chs 4 and 5.

82 Luca Enriques and others, ‘The Basic Governance Structure: Minority Shareholders and Non-Shareholder Constituencies’ in Kraakman and others (n 64) 92.

83 Tirole (n 67) 4.

84 For a more radical reconsideration of agency theory with a focus on the ‘public’ as a principal (and the state as its agent), see Dorothy S Lund, ‘Public Primacy in Corporate Law’ (2024) 47 Seattle U L Rev 365.

85 Easterbrook and Fischel, ‘The Corporate Contract’ (n 58) 37 (emphasis added).

86 Dorothy S Lund, ‘Toward a Dynamic View of Corporate Purpose Law’ (2023) ECGI Working Paper No 746/2023, 6–7.

87 Mariana Pargendler, ‘The Corporate Governance Obsession’ (2016) 42 J Corp L 359, 398–9, noting the scarcity of research on this topic.

88 Mariana Pargendler, ‘Corporate Law in the Global South: Heterodox Stakeholderism’ (2023) 47 Seattle U L Rev 535, 578.

89 Hayek (n 26).

90 Jensen (n 56) 246.

91 See eg Jill E Fisch and Steven D Solomon, ‘Should Corporations Have a Purpose?’ (2021) 99 Tex L Rev 1309, fn 148.

92 Roland Bénabou and Jean Tirole, ‘Individual and Corporate Social Responsibility’ (2010) 77 Economica 1, 1. For a classic exposition, see Nicholas Kaldor, ‘Welfare Propositions of Economics and Interpersonal Comparisons of Utility’ (1939) 49 The Economic Journal 549, 551. For a critical perspective questioning these premises, see William W Bratton and Michael L Wachter, ‘Shareholders and Social Welfare’ (2013) 36 Seattle U L Rev 489.

93 See Duncan Kennedy, ‘Legal Formality’ (1973) 2 JLS 351, 390 fn 57.

94 See Guido Calabresi, *Cost of Accidents: A Legal and Economic Analysis* (Yale UP 2008) 26, 31–3; Douglas G Baird and Thomas H Jackson, ‘Corporate Reorganizations and the Treatment of Diverse Ownership Interests: A Comment on Adequate Protection of Secured Creditors in Bankruptcy’ (1984) 51 U Chi L Rev 97, 103.

95 Bebchuck and Tallarita (n 1) 168–70.

96 Bebchuk and Tallarita recognise this as well. See ibid 96. Jensen states his preference for a Coasean private economy solution. See Jensen (n 56) 239.

97 See eg Eilís Ferran and Pedro Schilling de Carvalho, ‘Reconciling Shareholder Primacy and the Interests of People and Planet’ (2025) University of Cambridge Faculty of Law Research Paper No 825/2025, LQR (forthcoming); Leo E Strine Jr, ‘Our Continuing Struggle with the Idea That For-Profit Corporations Seek Profit’ (2012) 47 Wake Forest L Rev 135, 171; Rock (n 15) 394.

98 Bratton and Wachter, ‘Shareholder Primacy’s Corporatist Origins’ (n 72) 130.

99 Mayer, ‘Shareholderism versus Stakeholderism’ (n 1) 1870; Lund, ‘Public Primacy’ (n 84) 380. Similar arguments have been made already in Christopher D Stone, *Where the Law Ends: The Social Control of Corporate Behavior* (Harper & Row Publishers 1975) 194; Ralph Nader, Mark J Green and Joel Seligman, *Taming the Giant Corporation* (Norton 1976) 63–5; Einer Elhauge, ‘Sacrificing Corporate Profits in the Public Interest’ (2005) 80 NYU L Rev 733, 739.

100 Ann M Lipton, ‘Not Everything Is About Investors: The Case for Mandatory Stakeholder Disclosure’ (2020) 37 Yale J Reg 499, 505.

101 Fisch (n 60) 665; Strine, ‘Restoration’ (n 9) 421–3; Tirole (n 67) 25.

102 Michael E Levine and Jennifer L Forrence, ‘Regulatory Capture, Public Interest, and the Public Agenda: Toward a Synthesis’ (1990) 6 J L Econ & Org 167, 169.

103 For empirical research on lobbying, see Lee Drutman, *The Business of America is Lobbying* (OUP 2015).

104 Luigi Zingales, ‘Friedman’s Principle, 50 Years Later’ in Luigi Zingales, Jana Kasperkevic and Asher Schechter (eds), *Milton Friedman 50 Years Later* (Stigler Center 2020).

105 See eg Eric W Orts, ‘The Legitimacy of Multinational Corporations’ in Lawrence E Mitchell (ed), *Progressive Corporate Law* (Routledge 2019) 247, 253.

106 Adam Smith, *An Inquiry into the Nature and Causes of the Wealth of Nations by Adam Smith* (first published 1776, Liberty Classics 1976) 426.

107 Guido Palazzo and Andreas G Scherer, ‘Corporate Legitimacy as Deliberation: A Communicative Framework’ (2006) 66 Journal of Business Ethics 71.

108 Oliver Hart and Luigi Zingales, ‘Companies Should Maximize Shareholder Welfare Not Market Value’ (2017) 2 Journal of Law, Finance, and Accounting 247. In a recent study they also show, with Eleonora Broccardo, that shareholder engagement (voice) makes corporations behave more socially responsibly whereas divestments and boycotts (exit) may even reduce social welfare. See Eleonora Broccardo, Oliver Hart and Luigi Zingales, ‘Exit Versus Voice’ (2022) 130 J Polit Econ 3101.

109 They credit particularly Elhauge (n 99) 738–40. For others, see Gerald E Frug, ‘The Ideology of Bureaucracy in American Law’ (1984) 97 Harv L Rev 1276, 1314; Alex Edmans, *Grow the Pie: How Great Companies Deliver Both Purpose and Profit* (CUP 2020) 53.

110 Merritt B Fox and Menesh S Patel, ‘Common Ownership: Do Managers Really Compete Less?’ (2022) 39 Yale J Reg 136, 172 (and the references listed therein).

111 Hart and Zingales (n 108) 249.

112 See Dorothy S Lund, ‘Asset Managers as Regulators’ (2022) 171 U Pa L Rev 77; Pargendler, ‘The Corporate Governance Obsession’ (n 87) 398; Bratton and Wachter, ‘Shareholder Primacy’s Corporatist Origins’ (n 72) 525–56; Paddy Ireland, ‘Shareholder Primacy and the Distribution of Wealth’ (2005) 68 MLR 49; Marcel Kahan and Edward Rock, ‘Corporate Governance Welfarism’ (2023) 15 Journal of Legal Analysis 108, 111.

113 Thomas Donaldson and Lee E Preston, ‘The Stakeholder Theory of the Corporation: Concepts, Evidence, and Implications’ (1995) 20 Academy of Management Review 65.

114 ibid 71.

115 Bebchuck and Tallarita (n 1) 114.

116 Kenneth E Goodpaster, ‘Business Ethics and Stakeholder Analysis’ (1991) Business Ethics Quarterly 53.

117 See eg Michael Magill, Martine Quinzii and Jean-Charles Rochet, ‘A Theory of the Stakeholder Corporation’ (2015) 83 Econometrica 1685, 1689.

118 As many have argued, purely instrumental stakeholder theories are not conceptually or normatively distinguishable from shareholderism or ‘enlightened shareholder value’. See eg Eric W Orts and Alan Strudler, ‘The Ethical and Environmental Limits of Stakeholder Theory’ (2002) 12 Business Ethics Quarterly 215, 217; Bebchuck and Tallarita (n 1) 110–14; Edmans (n 109) 49–50.

119 As Tirole notes, ‘it is harder to measure the firm’s contribution to the welfare of employees, of suppliers, or of customers than to measure its profitability’. Tirole (n 67) 26.

120 Magill, Quinzii and Rochet (n 117) 1689.

121 ibid 1689.

122 Dirk Schoenmaker, Willem Schramade and Jaap Winter, ‘Corporate Governance Beyond the Shareholder and Stakeholder Model’ (2023) 16 Erasmus L Rev 16, 28. The impact accounting model, despite its focus on quantifiable values, also raises Rawlsian rights-based justifications. See Reinier De Adelhart Toolrop, Dirk Schoenmaker and Willem Schramade, ‘Decision Rules for Corporate Investment’ (2024) 12 International Journal of Financial Studies 24.

123 Schoenmaker, Schramade and Winter (n 122) 27. The conceptual basis for accounting-based framework was developed in John Elkington, *Cannibals with Forks: The Triple Bottom Line of 21st Century Business* (New Society Publishers 1998).

124 Margaret M Blair and Lynn A Stout, ‘A Team Production Theory of Corporate Law’ (1999) 85 Va L Rev 247, 315.

125 ibid 255.

126 ibid 315–19.

127 Colin Mayer, ‘The Future of the Corporation and the Economics of Purpose’ (2021) 58 Journal of Management Studies 887, 897.

128 Colin Mayer, *Prosperity: Better Business Makes the Greater Good* (OUP 2018) 38–40. See also The British Academy, *Principles for Purposeful Business* (2019).

129 Mayer, ‘The Future of the Corporation’ (n 127) 892.

130 The problem of internalising externalities has been considered a key function of property rules in economics for more than 50 years. See Harold Demsetz, ‘Toward a Theory of Property Rights’ (1967) 57 American Economic Review 347.

131 Mayer, ‘The Future of the Corporation’ (n 127) 897.

132 ibid 897.

133 ibid 892.

134 ibid 896, 889. For more developed property rights approaches with richer ethical foundations, see Donaldson and Preston (n 113) 83–5; Simon Deakin, ‘The Corporation as Commons: Rethinking Property Rights, Governance and Sustainability in the Business Enterprise’ (2012) 37 Queen’s LJ 339.

135 Thomas M Jones and Will Felps, ‘Stakeholder Happiness Enhancement: A Neo-utilitarian Objective for the Modern Corporation’ (2013) 23 Business Ethics Quarterly, 349, 358.

136 Ronald K Mitchell and others, ‘Stakeholder Agency and Social Welfare: Pluralism and Decision Making in the Multi-objective Corporation’ (2016) 41 Academy of Management Review 252, 266.

137 Beate Sjåfjell and Christopher M Bruner, ‘Corporations and Sustainability’. In Beate Sjåfjell and Christopher Bruner (eds), *The Cambridge Handbook of Corporate Law, Corporate Governance and Sustainability* (1st edn, CUP 2019) 11.

138 ibid 4.

139 Tirole (n 67) 5; Leo E Strine Jr, ‘Corporate Power Is Corporate Purpose II: An Encouragement for Future Consideration from Professors Johnson and Millon’ (2017) 74 Wash & Lee L Rev 1165, 1171; Bebchuck and Tallarita (n 1) 140.

140 Magill, Quinzii and Rochet (n 117) 1707–11.

141 According to them, corporate directors should be insulated ‘from the direct command and control of any of the groups that comprise the corporate team, including its shareholders’: Blair and Stout (n 124) 255 (emphasis added).

142 ibid 281.

143 ibid 282.

144 Mayer, *Prosperity* (n 128) 38.

145 Mayer, ‘The Future of the Corporation’ (n 127) 894; Mayer, *Prosperity* (n 128) 155.

146 See also The British Academy, *Principles for Purposeful Business* (n 128) 16.

147 ibid 19.

148 ibid.

149 ibid.

150 Beate Sjåfjell and Jukka Mähönen, ‘Corporate Purpose and the Misleading Shareholder vs Stakeholder Dichotomy’ (2022) 34 Bond LR 69, 83.

151 ibid 87.

152 ibid 88–90, 100.

153 Kershaw and Schuster (n 55) 482–3.

154 ibid 503.

155 Magill, Quinzii and Rochet (n 117).

156 Charles WL Hill and Thomas M Jones, ‘Stakeholder-Agency Theory’ (1992) 29 Journal of Management Studies 131. Mitchell and others also argue that corporations should be accountable to a broader range of individual and collective stakeholder preferences as revealed in the ‘intracorporate marketplace’: Mitchell and others, ‘Stakeholder Agency and Social Welfare’ (n 137).

157 Brett McDonnell, ‘Stakeholder Governance as Governance by Stakeholders’ (2024) 47 Seattle U L Rev 511.

158 Grant M Hayden and Matthew T Bodie, ‘A Democratic Participation Model for Corporate Governance’ (2025) 109 Minn L Rev 1579.

159 Henry Hansmann and Reinier Kraakman, ‘The End of History for Corporate Law’ (2001) 89 Geo LJ 439, 448.

160 Hansmann and Kraakman call this the ‘Fiduciary model’: ibid.

161 Mayer, ‘Shareholderism versus Stakeholderism’ (n 1); Mähönen and Sjåfjell (n 150).

162 Donaldson and Preston (n 113) 67. See also Edmans (n 109) 46: ‘Stakeholders are the end itself, rather than means to an end.’

163 Bertrand Russell, *History of Western Philosophy* (new edn, George Allen & Unwin 1961) 194.

164 See Engel (n 3) 29–32.

165 See eg Fisch and Solomon (n 91) 1333; Bebchuk and Tallarita (n 1) 98; Anant K Sundaram and Andrew C Inkpen, ‘Stakeholder Theory and “The Corporate Objective Revisited”: A Reply’ (2004) 15 Organization Science 370, 370.

166 For discussion of the various ethical bases of normative stakeholderism, see Elisabeth Garriga and Domènec Melé, ‘Corporate Social Responsibility Theories: Mapping the Territory’ (2004) 53 Journal of Business Ethics 51, 60–1. Social contract theory is developed in T Donaldson and TW Dunfee, *Ties That Bind: A Social Contracts Approach to Business Ethics* (Harvard Business School Press 1999).

167 Tirole (n 67) 24.

168 See Jones and Felps (n 135).

169 See Ronald K Mitchell, Bradley R Agle and Donna J Wood, ‘Toward a Theory of Stakeholder Identification and Salience: Defining the Principle of Who and What Really Counts’ (1997) 22 Academy of Management Review 853, identifying 27 different definitions of stakeholders in literature.

170 Freeman defines stakeholders as ‘any group or individual who can affect or is affected by the achievement of the organisation’s objectives’: R Edward Freeman, *Strategic Management: A Stakeholder Approach* (CUP 1984).

171 Donaldson and Preston argue that relevant stakeholders should be identified based on ‘their legitimate interest in the corporation’: Donaldson and Preston (n 113) 76.

172 Mayer, *Prosperity* (n 128) 3.

173 Schoenmaker, Schramade and Winter (n 122) 33. Edmans also argues that material stakeholders are business-specific and identifying them should fall within directors’ judgment: Edmans (n 109) 71–3.

174 Fisch and Solomon (n 91) 1337.

175 Tirole (n 67) 26; Hart (n 60) 303.

176 Jensen (n 56) 237; Hansmann and Kraakman (n 159) 448.

177 Bebchuk and Tallarita (n 1); Ferran and De Carvalho (n 97). For a recent critical analysis of this view, see Aneil Kovvali, ‘Stark Choices for Corporate Reform’ (2023) 123 Colum L Rev 693.

178 Magill, Quinzii and Rochet (n 117) 1707–11.

179 Schoenmaker, Schramade and Winter (n 122) 28.

180 ibid 34–5.

181 ibid 34.

182 ibid.

183 Donaldson and Preston (n 113) 87.

184 Mayer, ‘Shareholderism versus Stakeholderism’ (n 1) 1877.

185 The British Academy, *Policy & Practice* (n 2).

186 ibid.

187 See Mayer, *Prosperity* (n 128) 39; Beate Sjåfjell and others, ‘Shareholder Primacy: The main Barrier to Sustainable Companies’ in Beate Sjåfjell and Benjamin J Richardson (eds), *Company Law and Sustainability* (CUP 2015) 95, 88, arguing that the ‘business judgement rule and the discretionary authority it vests in boards is a possible friend not foe of sustainability’.

188 Stone (n 99) 80, 114–15.

189 Elhauge (n 99) 751–4.

190 E Merrick Dodd Jr, ‘Is Effective Enforcement of the Fiduciary Duties of Corporate Managers Practicable?’ (1935) 2 U Chi L Rev 194, 203–4.

191 Lee (n 26) 568–70.

192 Sjåfjell and others, ‘Shareholder Primacy’ (n 187) 94.

193 Mayer, *Prosperity* (n 128) 39.

194 See Fritz Scharpf, ‘Legitimacy in the Multilevel European Polity’ (2009) 1 European Political Science Review 173, 174–5.

195 ibid 174.

196 Jeffrey Moriarty, ‘The Connection Between Stakeholder Theory and Stakeholder Democracy: An Excavation and Defense’ (2014 53 Business & Society 820.

197 David Millon, ‘Communitarians, Contractarians, and the Crisis in Corporate Law’ (1993) 50 Wash & Lee L Rev 1373, 1378 fn 29.

198 As Elster notes, this requirement is a fundamental tenet of social choice theory. See Elster (n 38) 6.

199 Andreas G Scherer, Guido Palazzo and Dorothée Baumann, ‘Global Rules and Private Actors: Toward a New Role of the Transnational Corporation in Global Governance (2006) 16 Business Ethics Quarterly 505, 520.

200 This view has a long pedigree, particularly in American legal and political thought. See Dalia Tsuk, ‘From Pluralism to Individualism: Berle and Means and 20th-Century American Legal Thought’ (2005) L & Soc Inquiry 179, 184.

201 See Paul Tucker, ‘Unelected Power: The Quest for Legitimacy in Central Banking and the Regulatory State’ (Princeton UP 2019).

202 Gadinis and Havasy (n 6).

203 Article 13 of Directive (EU) 2024/1760 of the European Parliament and of the Council of 13 June 2024 on corporate sustainability due diligence [2024] OJ L2024/1760 (EU CSDDD).

204 EU CSDDD, rec 65.

205 Directive (EU) 2022/2464 of the European Parliament and of the Council of 14 December 2022 amending Regulation (EU) No 537/2014, Directive 2004/109/EC, Directive 2006/43/EC and Directive 2013/34/EU, as regards corporate sustainability reporting [2022] OJ L322 (EU Corporate Sustainability Reporting Directive).

206 EU Corporate Sustainability Reporting Directive, rec 14.

207 Giandomenico Majone, ‘Regulatory Legitimacy’ in Giandomenico Majone (ed), *Regulating Europe* (Taylor & Francis Group 1996) 291. See also Tucker (n 201) ch 15.

208 See, however, Accountable Capitalism Act (S 3348, 115th Cong (2018)) in the United States, which proposed the introduction of a federal chartering requirement for large companies. For an early articulation of this idea, see Ralph Nader, ‘The Case for Federal Chartering’ in Mark J Green and Ralph Nader (eds), *Corporate Power in America* (Grossman Publishers 1973) 85–9.

209 Kent Greenfield, ‘Ultra Vires Lives! A Stakeholder Analysis of Corporate Illegality (with Notes on How Corporate Law Could Reinforce International Law Norms)’ (2001) 87 Va L Rev 1279, 1282.

210 Stavros Gadinis, ‘Social Business Judgment’ Bus Law (forthcoming) <https://ssrn.com/abstract=5284487> (noting at 51 that ‘the scope of review would focus on process rather than substance’).

211 Majone, ‘Europe’s “Democratic Deficit”’ (n 37) 28.

212 ibid 11–13.

213 Elster (n 38) 12.

214 Palazzo and Scherer (n 107).

215 ibid.

216 See eg Risse (n 32); Jen Steffek, Claudia Kissling and Patrizia Nanz (eds), *Civil Society Participation in European and Global Governance. A Cure for the Democratic Deficit?* (Palgrave Macmillan 2008).

217 See eg Elster (n 38) 6.

218 Julia Black, ‘Proceduralizing Regulation: Part I’ (2000) 20 OJLS 597, 608.

219 Julia Black, ‘Proceduralizing Regulation: Part II’ (2001) 21 OJLS 33, 34.

220 Black, ‘Part I’ (n 218) 598.

221 The EU Corporate Sustainability Reporting Directive, rec 19.

222 Bhupinder S Chimni, ‘Co-option and Resistance: Two Faces of Global Administrative Law’ (2005) 37 NYU J Int’l Law & Pol 799.

223 Susan Marks, ‘Naming Global Administrative Law’ (2004) 37 NYU J Int’l Law & Pol 995, 997.

224 Engel (n 3) 18, 85; Romano (n 4) 924.

225 Philip G Cerny, ‘Globalization and the Erosion of Democracy’ (1999) 36 European Journal of Political Research 1; Dani Rodrik, ‘How Far Will International Economic Integration Go?’ (2000) 14 Journal of Economic Perspectives 177.

226 Easterbrook and Fischel, *The Economic Structure of Corporate Law* (n 54) 37.

227 Kershaw and Schuster (n 55) 521.

